# Tristetraprolin/ZFP36 Regulates the Turnover of Autoimmune-Associated HLA-DQ mRNAs

**DOI:** 10.3390/cells8121570

**Published:** 2019-12-04

**Authors:** Laura Pisapia, Russell S. Hamilton, Federica Farina, Vito D’Agostino, Pasquale Barba, Maria Strazzullo, Alessandro Provenzani, Carmen Gianfrani, Giovanna Del Pozzo

**Affiliations:** 1 Institute of Genetics and Biophysics “Adriano Buzzati Traverso” CNR, Via Pietro Castellino, 111, 80131 Naples, Italy; laurapisapia@virgilio.it (L.P.); federica.farina@igb.cnr.it (F.F.); pasquale.barba@igb.cnr.it (P.B.); maria.strazzullo@igb.cnr.it (M.S.); 2 Centre for Trophoblast Research, Department of Physiology, Development and Neuroscience, University of Cambridge, Downing Site, Cambridge CB2 3DY, UK; rsh46@cam.ac.uk; 3 Centre for Cellular, Computational and Integrative Biology-CIBIO, University of Trento, via Sommarive 9, 38123 Trento, Italy; vito.dagostino@unitn.it (V.D.); alessandro.provenzani@unitn.it (A.P.); 4 Institute of Biochemistry and Cell Biology-CNR, Via Pietro Castellino, 111, 80131 Naples, Italy; c.gianfrani@ibp.cnr.it

**Keywords:** celiac disease, Human Leukocyte Antigen (HLA), RNA binding protein, RNA stability, RNA structure

## Abstract

HLA class II genes encode highly polymorphic heterodimeric proteins functioning to present antigens to T cells and stimulate a specific immune response. Many HLA genes are strongly associated with autoimmune diseases as they stimulate self-antigen specific CD4^+^ T cells driving pathogenic responses against host tissues or organs. High expression of HLA class II risk genes is associated with autoimmune diseases, influencing the strength of the CD4^+^ T-mediated autoimmune response. The expression of HLA class II genes is regulated at both transcriptional and post-transcriptional levels. Protein components of the RNP complex binding the 3′UTR and affecting mRNA processing have previously been identified. Following on from this, the regulation of HLA-DQ2.5 risk genes, the main susceptibility genetic factor for celiac disease (CD), was investigated. The DQ2.5 molecule, encoded by HLA-DQA1*05 and HLA-DQB1*02 alleles, presents the antigenic gluten peptides to CD4^+^ T lymphocytes, activating the autoimmune response. The zinc-finger protein Tristetraprolin (TTP) or ZFP36 was identified to be a component of the RNP complex and has been described as a factor modulating mRNA stability. The 3′UTR of CD-associated HLA-DQA1*05 and HLA-DQB1*02 mRNAs do not contain canonical TTP binding consensus sequences, therefore an in silico approach focusing on mRNA secondary structure accessibility and stability was undertaken. Key structural differences specific to the CD-associated mRNAs were uncovered, allowing them to strongly interact with TTP through their 3′UTR, conferring a rapid turnover, in contrast to lower affinity binding to HLA non-CD associated mRNA.

## 1. Introduction

HLA class II genes are the main risk factor associated with several autoimmune diseases (https://www.niehs.nih.gov/health/topics/conditions/autoimmune/index.cfm). These genes encode heterodimers presenting antigenic self-peptides that are expressed on the surface of antigen presenting cells (APC) such as dendritic cells, B lymphocytes, and macrophages, with the role of presenting antigens to CD4^+^ T cells. Following self-antigen presentation, cognate CD4^+^ T cells are activated and proliferate determining the inflammatory response and organ damage. The expression of the HLA class II molecules influences the concentration of HLA-antigen complexes on APC and, consequently, the strength of CD4^+^ T cells autoimmune response.

The expression of HLA class II genes is strictly regulated at the transcriptional level by a highly conserved regulatory module, situated 150–300 base pairs upstream of the transcription-initiation site [1]. This regulatory module interacts with several DNA binding factors, which, in turn, bind the HLA class II trans-activator CIITA [2]. The expression of HLA class II molecules has been demonstrated to be regulated through a mechanism that coordinates transcription and processing in the context of an “MHCII RNA operon”, a functional unit through which the UTRs of different MHC class II transcripts bind the same RNP complex [3,4]. Two RNA binding proteins, EBP1, ErbB3 binding protein [5], and NF90 (Nuclear Factor 90) [6] were found to interact with stem-loop secondary structures at 5′ and 3′ UTRs of HLA-DRA and HLA-DRB1 mRNAs encoding for HLA-DR heterodimer, whose expression is downregulated by the depletion of both proteins. The discovery of a specific protein complex regulating the expression of HLA DR transcripts opens up the possibility that HLA-DQ, another isotype, may be regulated under the same mechanism. The HLA-DQ molecule is involved in self-antigen presentation, typical of many autoimmune pathologies.

The HLA-DQ2.5 molecule is strongly associated with celiac disease (CD), a gluten-triggered disorder [7] and type 1 diabetes (T1D) [8], two autoimmune pathologies in which the main genetic predisposing factor is conferred by HLA-DQ2.5 encoding genes. More than 95% of CD patients express the HLA-DQ2.5 molecule, while the majority of remaining affected subjects carry the HLA-DQ8 molecule. In T1D patients, the presence of the HLA-DQ2.5 molecule is less frequent than HLA-DQ8, and 3.5–10% of individuals are affected by co-morbidities of CD and T1D. The surface expression of DQ2 and/or DQ8 molecules, on antigen presenting cells (APC), determines the level of self/gluten-antigen presentation and, consequently, the magnitude of the pathogenic autoimmune CD4^+^ T cell response leading to organ damage. It was recently demonstrated that DQA1*05 and DQB1*02 genes are more highly expressed than other non-CD-associated alleles in APC from CD patients carrying the DQ2-DR3 genotype in heterozygosis [9]. DQA1*05 and DQB1*02 mRNAs were found to show a similar stability, although lower with respect to other DQA1*01 and DQB1*05 non-disease associated transcripts. In this study, the differential expression of HLA class II transcripts was investigated to ascribe a specific protein of the ribonucleoprotein complex to preferentially bind the autoimmune-associated transcripts. 

TTP (ZFP36) is a RNA binding protein that preferentially binds to AU-rich regions contained in the 3′UTR of target genes. TTP functions by destabilizing mRNAs encoding for oncogenes, cytokines (as TNFα), and chemokines involved in the inflammatory processes, by favoring their degradation and/or preventing their efficient translation [10,11]. Studies in TTP KO mice demonstrate that TTP not only regulates the primary immune cellular response to innate stimuli, but also regulates the expression of transcripts involved in the secondary response of fibroblasts/macrophages to the TNFα stimulation [12,13]. Here, the TTP protein is determined to be a component of the MHC operon and is involved in the regulation of the processing of the HLA-DQ genes.

## 2. Results 

### 2.1. TTP Binds to CD—Associated and Non-Associated Transcripts

To analyze the interaction of RNA binding proteins with 3′UTR of DQA1* and DQB1* transcripts, DNA templates were synthesized for in vitro transcription to obtain the different riboprobes. The cDNA of immortalized B-cells (B-LCL#5) obtained from a celiac patient carrying DQA1*01-DQA1*05/DQB1*02-DQB1*05 genotype [9] was used to synthetize the two riboprobes corresponding to 3′UTR of DQA1*05 (3DQA105 riboprobe) and DQB1*02 (3DQB102 riboprobe) mRNAs, encoding the DQ2.5 molecule associated with CD. The cDNA from HOM-2 cell line, carrying the homozygous DQA1*01/DQB1*05 genotype, was used for the synthesis of two riboprobes corresponding to the 3′UTR of DQA1*01 (3DQA101) and DQB1*05 (3DQB105) mRNAs, encoding DQ5 molecule not associated with disease.

**Figure 1:**
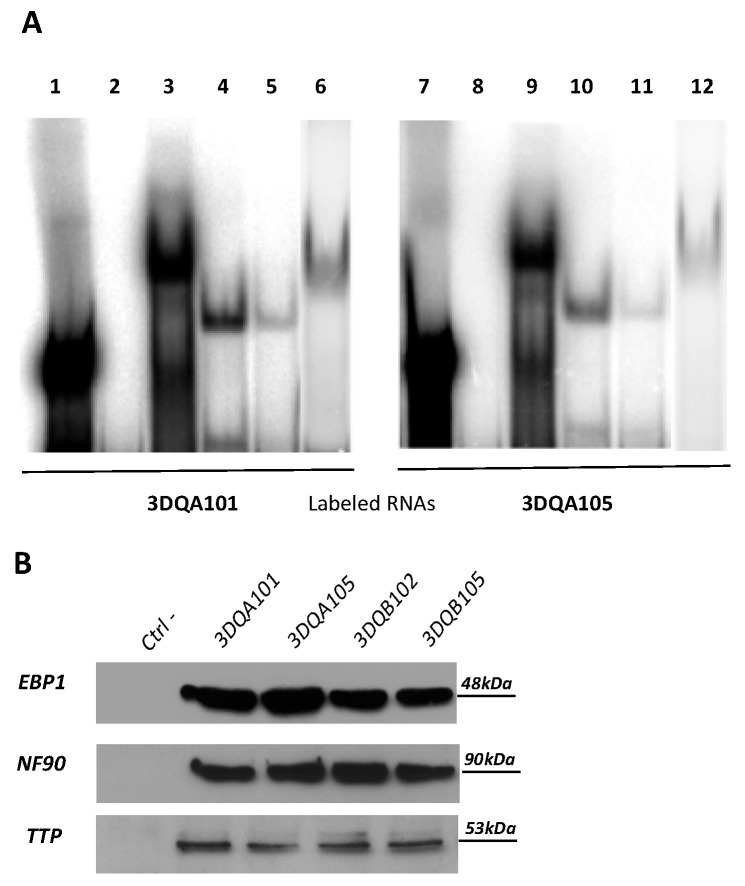
RNA binding proteins interaction. (**A**) REMSA experiments performed using 3DQA101 (Lane 1) and 3DQA105 (Lane 9) riboprobes. Lanes 2 and 8 show the digestion of riboprobes with T1 RNase. The binding of DQA101 with M14 extract is in Lane 3 and with B-LCL#5 extract in Lane 6. The binding of 3DQA105 with M14 extract is in Lane 9 and with B-LCL#5 extract in Lane 12. The interaction of 5 and 1 µg of rTTP with 3DQA101 is shown in Lanes 4 and 5 and with 3DQA105 in Lanes 10 and 11. (**B**) Western blot analysis of biotin pull-down assay carried by using 3DQA101, 3DQA105, 3DQB102, and 3DQB105 riboprobes. The antibodies used for the immunoblot were anti-NF90, anti-TTP, and anti-EBP1. Molecular weights are as indicated.

REMSA experiments were performed. Figure 1A shows the interaction of 3DQA101 (Lanes 3 and 6) and 3DQA105 (Lanes 9 and 12) riboprobes to S100 cytoplasmic extracts of M14 and B-LCL#5, respectively. It was also demonstrated that recombinant TTP (rTTP) interacts with either 3DQA101 (Lanes 4 and 5) or 3DQA105 (Lanes 10 and 11) riboprobes. 

To confirm that this complex includes the same proteins previously identified, a pull-down assay was performed (Figure 1B). In vitro transcribed biotinylated riboprobes, 3DQA101, 3DQA105, 3DQB102, and 3DQB105, were incubated with S100 cytoplasmic extract of B-LCL#5 and, after pull-down with streptavidin coated beads, proteins interacting with biotinylated riboprobes were detected by Western blot analysis. By using anti-NF90, anti-EBP1 and anti-TTP, it was demonstrated that all three proteins bind to the 3′UTRs of DQA1* and DQB1* mRNAs. In conclusion, NF90 and EBP1, previously identified as components of the complex interacting with 3′UTRs of DRA and DRB1 mRNA [3,4], were confirmed to interact with DQ riboprobes while, for the first time, TPP was identified to be a component of the RNP complex binding the HLA-DQA1*01, DQA1*05, DQB1*02, and DQB1*05 mRNAs.

### 2.2. Analysis of DQA1* and DQB1* 3′UTR Sequences

**Figure 2:**
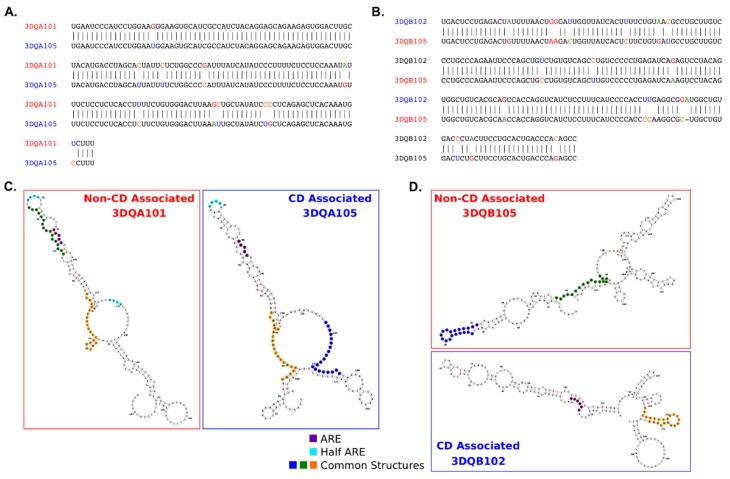
Sequences and structures comparison between 3′UTR of DQA1* and DQB1* genes. (**A**) Comparison of the 3′UTR sequences for the DQA1*01 and DQA1*05 alleles indicates 94.1% sequence identity. There is a slightly higher proportion of GC content in the DQA1*01 allele (0.46) as compared to DQA1*05 (0.44). (**B**) The DQB1*05 and DQB1*02 alleles show a lower sequence identity (92.3%), but identical GC proportions (0.55). (**C**,**D**) RNA secondary structure prediction reveals the sequence differences between alleles impacts the minimum-free energy folding (RNAfold). Common structural motifs from FOLDALIGN are mapped onto the structures as well as canonical ARE (AUUUA/AUUUUA) and half-ARE motifs (e.g., UAUU).

TTP interacts with transcripts via the specific AU-rich AREs in the 3′UTR of target genes and is influenced by the folding of the mRNA itself. Therefore, it was investigated whether differences in the nucleotide sequences may affect RNA folding, the distribution of AU rich elements, and their secondary structure accessibility along the 3′UTR. Firstly, by comparing the nucleotide sequences of the 3′UTRs of DQA1*05 and DQB1*02, CD-associated transcripts, with the 3′UTRs of DQA1* 01 and DQB1*05, non-CD-associated transcripts and to highlight the differences in Figure 2A,B. In the DQA1* alleles, a 94.1% identity was observed and a small difference in the overall GC content with the DQA1*01 allele at 0.46 as compared to DQA1*05 at 0.44 (Figure 2A). In the DQB1* alleles, the sequence identity between 3DQB102 and 3DQB105 riboprobes was lower, at 92.3%, despite the alleles sequence having identical GC content (0.55) (Figure 2B). The sequence differences between alleles are likely to influence the secondary structures of the RNAs, minimum-free energy (MFE) structure predictions were performed (Figure 2C,D), and mapped on the canonical ARE (AUUUA/AUUUUA) and half-ARE (UAUU) motifs associated with TPP binding [11,14]. A longer length ARE sequence has been defined as WWAUUUAWW [13](W represents A or U), however this motif is not present in the DQA1* or DQB1* 3′UTRs. The canonical ARE and half-ARE motifs are present in both 3DQA101 and 3DQA105 riboprobes. In contrast, the ARE motif is only found in the 3DQB102 riboprobe (Figure 2D), suggesting the potentiality for TTP to bind to CD-associated transcripts. Alignments of the DQA1* and DQB1* alleles with their mouse homologs, H2-Aa and H2-Ab1 (Figure S1), reveal sequence identities of DQA1* and DQB1* with their mouse homologs to be <60% and <40%, respectively, indicating a low level of conservation of the UTRs across species.

To assess RNA secondary structure stability between the DQA1* and DQB1* pairs, ensembles of statistically sampled structures using Sfold [15] were calculated (Figure S1). The ensembles represent the full range of structures a sequence can adopt, not just those with the lowest MFE, highlighting substructures that remain constant across all structure conformations. MFE prediction makes the assumptions that there is a single rigid structure and that the energy parameters are complete. However, it is likely that mRNAs are more dynamic and can adopt multiple conformations depending on their environment and presence of RNA binding proteins. Across the predicted ensemble structures the conserved substructures co-localize with the ARE and half-ARE sequence motif positions in the 3DQA101, 3DQA105, and 3DQB105 mRNAs. The 3DQB102 ARE is outside of a predicted stable substructure, indicating it is likely to be more flexible and adopt a range of structural conformations. The proportion of the 3DQB102 structure with predicted conserved substructures between ensembles and minimum-free energy predictions, and those from FOLDALIGN (see below), is lower than in the other alleles and, importantly, does not contain AU–rich elements, indicating that TTP may bind in regions with low structural stability. 

Structural motif searches between pairs of 3′UTRs with FOLDALIGN [16] determine if there are any common structural motifs (defined by sequence and secondary structure) that could confer an RNA Operon-like module as previously found [4]. The presence of a common motif present in different structures could indicate a common RNA-binding protein site. The identified common structural motifs have been mapped onto the structures in Figure 2C,D. Structures common to the DQA1* and DQB1* pairs were found and support the hypothesis this could enable them to co-bind in an “RNA Operon”, as has been previously demonstrated for the HLA-DR alleles [4].

**Figure 3:**
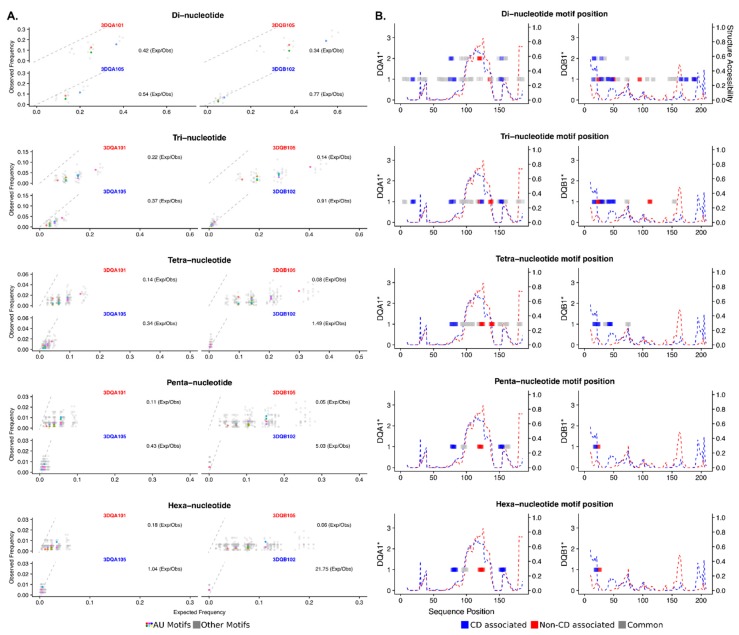
AU-rich motif analysis of the DQA1* and DBQ1* genes reveals key differences. (**A**) All possible di-, tri-, tetra-, penta- and hexa-mers are assessed for their observed frequencies against their expected frequencies. AU-rich motifs are colored, non-AU rich in grey. All AU-rich kmer motifs have higher enrichments in the CD associated DQA1*05 and DQB1*02 alleles as compared to the non-CD associated DQA1*01 and DQB1*05 alleles. (**B**) Mapping the AU-rich motifs to their position within the 3′UTR sequences reveals a clear enrichment in AU-motifs throughout the CD associated alleles DQA1*05 and DQB1*02 (Blue) as compared to the non-CD associated DQA1*01 and DQB1*05 alleles (Red). Nested motifs are indicated on the y-axis; for example, two overlapping motifs will show a score of 2. A structure accessibility score indicates the likelihood the RNA single stranded, an absence of base-pairing, across its length. A score of 1.0 indicates the structure is single stranded.

A more extensive AU rich motif search was undertaken to determine if there are further, possibly weaker, TPP binding sites in the riboprobes and whether there are differences between the CD and non-CD associated allele pairs. A kmer analysis at a range of motif lengths (di, tri, tetra, penta, and hexa nucleotides) shows a clear pattern of enrichment of AU containing motifs in the CD associated alleles as compared to the non-disease associated across all kmer lengths (Figure 3A). Mapping the AU rich kmers onto the riboprobe sequences reveals the CD associated alleles contain more AU rich sites located across the full length of the sequence and therefore are likely to confer more TPP binding through these AU motifs. Individual AU rich kmers are also more commonly seen in clusters together in close proximity in the CD associated alleles than those non-disease associated (Figure 3B).

The secondary structure context of the AU rich kmers is likely to influence the ability of TPP to bind in a sequence specific manner. Therefore, RNAplfold was used to calculate a secondary structure accessibility score, indicating the probability of whether the RNA is likely to be single stranded along its length. In the DQA1* alleles, the AU motifs are more prevalent in the CD-associated allele; however, they tend to be localized in regions with a lower probability of being single-stranded than the non-disease associated allele. In DQB1*, the AU motifs in the CD-associated alleles are not only more common but also colocalized with single-stranded regions, in contrast the AU motifs in the non-disease allele are not in single-stranded regions.

In conclusion, the in silico analysis predicts that TTP binds to HLA-DQA1 and HLA-DQB1 mRNAs and that the secondary structures adopted by the alleles facilitate the interaction of TTP with the 3′UTR of CD-associated mRNAs. According to the canonical role of TTP, this binding should confer lower stability to transcripts respect to non-CD associated mRNAs.

### 2.3. Knockdown of TTP Affects the Expression Level of DQA1 and DQB1 Transcripts

**Figure 4:**
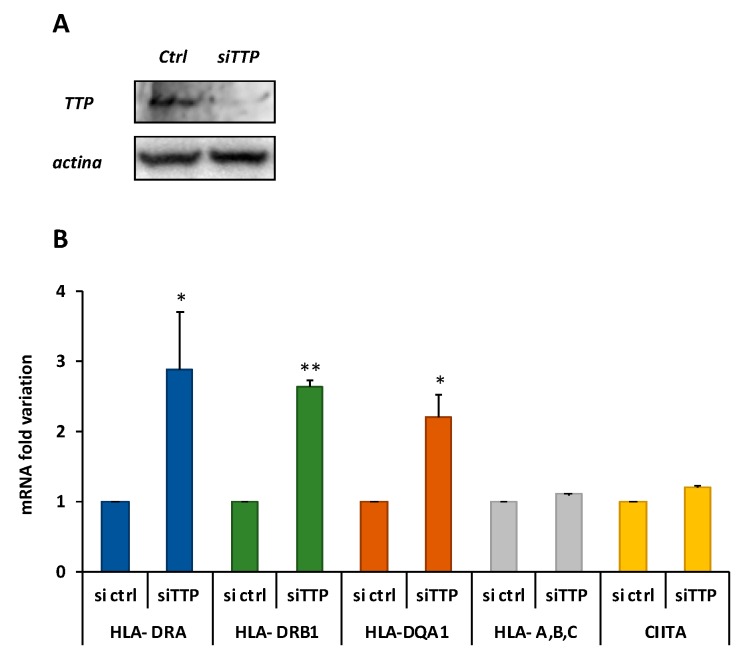
TTP knockdown in M14. (**A**) Western blot performed with anti-TTP for the assessment of protein depletion 48 h after silencing with siCtlr or siTTP (**B**) Fold variation of DRA, DRB1, and DQA1 mRNAs. HLA-a,b,c are the class I mRNAs, CIITA is the HLA class II transcriptional transactivator.

**Figure 5:**
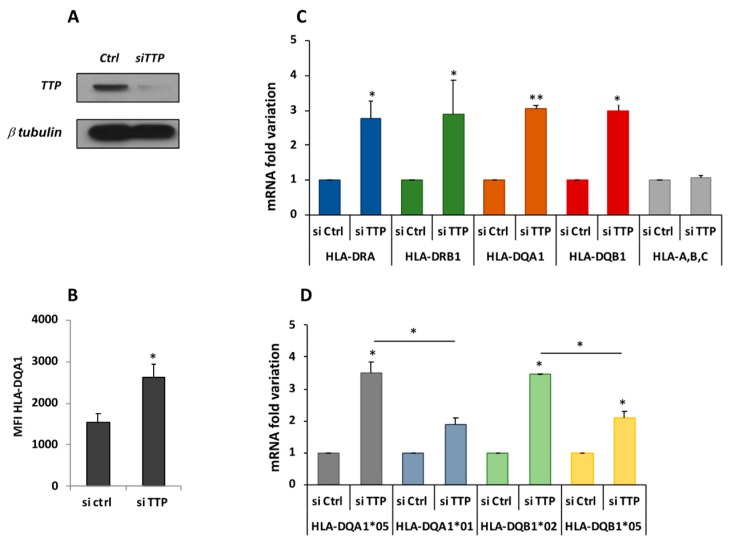
TTP knockdown in B-LCL#5. (**A**). Western blot performed with anti-TTP for the assessment of protein depletion 48 h after silencing with siCtlr or siTTP nucleofection. (**B**) Cytofluorimetric analysis of HLA-DQ surface expression, reported as fold change of MFI (Mean Fluorescence Intensity). (**C**) Fold variation of DRA, DRB1, DQA1, DQB1, and HLA-A, -B, and -C (class I) mRNAs. (**D**) Fold variation of DQA1*01, DQA1*05, DQB1*02, and DQB1*05 mRNAs.

It was previously demonstrated that there is differential expression of CD-associated transcripts, DQA1*05 and DQB1*02, and lower half-lives in respect to non-CD associated DQ transcripts in B-LCL#5 cells [9]. To investigate if the TTP protein is responsible for the DQ mRNAs turnover, the protein was depleted by specific siRNAs transfection in M14 and B-LCL#5 cell lines. The knockdown of TTP was first assayed in M14, 48 h after transfection by Western blot with anti-TTP (Figure 4A), in which we observed 80% depletion. When we measured the mRNA by qRT-PCR, we found 2–3-fold increase of DRA, DRB1*, and DQA1* transcripts (Figure 4B). As a control of the knockdown specificity, no variations were found in the expression of two groups of genes related to this system, the HLA-A, -B, and -C class I genes and CIITA, the HLA class II transcriptional activator. In parallel, a TTP knock-down was performed in B-LCL#5 cells by specific siRNA nucleofection, and the protein depletion after 48 h by Western blot was confirmed (Figure 5A). 

The TTP knockdown affects the phenotype of the cells and, by flow cytometry analysis, higher HLA-DQ surface expression was observed (Figure 5B). To evaluate whether the DQ increase corresponds to the HLA-DQ mRNAs variation qRT-PCR was carried out, revealing a 2.7- and 2.9-fold increase of DRA and DRB1* mRNAs, respectively. Total DQA1* and DQB1* mRNAs showed a three-fold increase compared to the control (siCtlr) and in respect to the HLA class I mRNAs (Figure 5C). To investigate if TTP affects the expression of each mRNAs, DQA1*01, DQA1*05, DQB1*02, and DQB1*05 mRNAs were specifically quantified. This confirmed that DQA1*05 and DQB1*02 have a higher expression than DQA1*01 and DQB1*05 (Figure 5C) as previously demonstrated [9]. However, when comparing the amount of different messengers after TTP knockdown, it was observed that DQA1*05 and DQB1*02 mRNAs showed a greater and significant increment (3.5-fold increase), in respect to DQA1*01 and DQB1*05 mRNA (two-fold increase, Figure 5D).

In conclusion, the depletion of TTP protein, as well as interaction with 3′UTR, determines an increment of the CD-associated DQA1*05 and DQB1*02 mRNAs greater than that of the non-CD-associated DQA1*01 and DQB1*05 mRNAs.

## 3. Discussion 

In many autoimmune diseases, HLA class II molecules are expressed on APCs and present self-antigens to CD4^+^ T cells. The magnitude of the CD4^+^ T cell activation and proliferation is related not only to the nature of cognate epitopes, but also to the amount of the antigen-HLA class II complexes expressed on the surface of APCs [17]. In this respect, the expression level of HLA class II molecules that restricts the antigenic responses is very important, particularly in autoimmune diseases as the HLA class II encoding genes represent the main genetic risk factor associated with these pathologies.

Previously, it was demonstrated that there are similar amounts of DQ2.5 molecules in APCs of CD patients of either heterozygous or homozygous for DQ2.5 risk genes [9]. Consequently, DQ2.5 heterozygous or homozygous APCs induced a similar strength of functional immune response by gluten-reactive CD4^+^ T cells. The equivalent surface density of DQ2.5 heterodimer on cells from homozygous and heterozygous CD patients has been linked to the marked difference in expression of DQA1*05 and DQB1*02 risk genes, in respect to the reduced expression of non-disease associated DQA1*01 and DQB1*05 alleles, in the DQ2.5 heterozygous genotype. This difference in the quantity of transcripts might be explained by haplotype-specific transcriptional regulation or allele-specific mRNA processing [17]. Indeed, the transcripts of both CD-associated DQA1*05 and DQB1*02 genes showed a similar decay kinetic (3 h half-life), but much lower in respect to non-CD-associated DQA1*01, DQB1*03, and DQB1*05 mRNAs (4 h half-life) [9]. This difference in the transcript turnover led to the hypothesis that a protein complex might differentially bind the autoimmune-associated messengers, and therefore modulate their decay.

The analysis of 3′UTR of CD-associated DQA1*05 and DQB1*02, and non-CD associated DQA1*01 and DQB1*05 alleles revealed several differences in GC content and in canonical ARE (AUUUA) and half-ARE (UAUU) motifs in addition to base differences that influence the secondary structures and binding sites of the RNAs. The predicted RNA structures show no differences in the motifs containing common substructures between DQA1* mRNAs, but lower structural stability for DQB1*02. The lower stability of this transcript influences the half-life of DQA1*05, as the turnover of the two mRNAs is co-regulated. In other words, the amount of a messenger establishes the quantity of its partner messenger coupled in the same RNP complex [4,17]. REMSA experiments confirm the in silico findings, revealing the interaction of cytoplasmic extracts with 3′UTR of DQA1*01 and DQA1*05 transcripts. Importantly, this indicates that the RNA binding proteins included in the complex are constitutively expressed by either professional (B-LCL) and/or non-professional antigen presenting cells (M14). Moreover, in these experimental conditions, the 3′UTR of DQB1*02 and DQB1*05 RNAs do not show binding with cytoplasmic extracts. The absence of binding with beta riboprobes may be caused by the radiolabeling of the 3′UTRs affecting their secondary structures, therefore the RNA–protein interaction was investigated by an end-labeled desthiobiotin based pull-down method that does not interfere with the RNA structure. Indeed, following the efficient enrichment of the protein–RNA complexes, assessed by immunoblot, the interaction of two RNA binding proteins, EBP1 and NF90, previously identified in the complex with of DRA and DRB1* 3′UTR [3,4], with four 3DQA105, 3DQA101, 3DQB102 and 3DQB105 riboprobes analyzed in this work was confirmed.

To investigate the different turnover of messengers, the interaction of TTP (ZNFP36), a zinc finger protein interacting with AU-rich elements (ARE)-containing mRNAs promoting rapid cytoplasmic decay through acceleration of deadenylation rate was investigated [18]. The interaction of recombinant TTP to the 3′UTR of all transcripts was assayed by REMSA and/or pull-down. In addition, to verify if TTP protein affects the stability, the depletion by specific siRNA was undertaken, and, as expected, an increase of HLA class II mRNAs was observed, in both cell lines, while HLA class I and CIITA mRNAs were unaffected. Notably, in B-LCL#5, TTP depletion results in a 3.5-fold increase of DQA1*05 and DQB1*02 mRNAs and two-fold increase of DQA1*01 and DQB1*05 mRNAs. These results clearly indicate a stronger binding of TTP and a consequent lower stability of CD-associated messengers in respect to non-CD associated alleles. 

Tristetraprolin has previously been shown to bind via canonical ARE (AUUUA) motifs, present in the upstream region of 3DQA101 and 3DQA105 riboprobes. In addition to the ARE motifs, there are also half-ARE (UAUU) motifs present in the 3DQA101 (two copies) and 3DQA105 riboprobes (Figure 1). More recently, TTP was found to bind to AU-rich sequences [11,14], and through sequence analysis an enrichment of AU-rich motifs with TPP binding potential in the CD-associated alleles as compared to the non-CD associated was demonstrated. These motifs are located throughout the sequences of the CD-associated alleles in contrast the non-CD-associated alleles where they form a cluster within positions 90–160 (approximate) (Figure 2). The sequence and AU-rich motif distribution differences between the DQA1 and DQB1 alleles is also likely to have an impact on the secondary structures of the RNAs and their accessibility to TPP binding. The analysis of the ensembles of predicted structures indicates that, in addition to the sequence motifs, there are differences in the structure stabilities contributing to the observed differences of TPP binding affinities (Figure S1). Across all the secondary structure conformations (ensembles) that can be adopted by each of the RNA alleles, it was observed that ARE motifs are co-located with the substructures present across all ensemble structures, thus indicating that they are present in the more stable sections of the structures. In the case of 3DQB102, an ARE is not found in the 3DQB105 riboprobe, indicating that TPP binding may higher affinity in 3DQB102, although this ARE is outside of the more stable substructures. In conclusion, the in silico analysis demonstrates that CD-associated mRNAs can strongly interact with TTP through their 3′UTR, conferring a more rapid mRNA turnover.

mRNAs controlling inflammation in mice were recently investigated, where it has been reported that no significant binding of TTP has been detected to H2-Aa and H2Ab1 mRNA, the mouse HLA-DQ-homologous transcripts [13]. However, there is low sequence identity between human and mouse HLA-DQ 3′UTRs (see Figure S2). Therefore, the function of TTP as an RNA-binding protein is likely to be more context-dependent than previously described in the literature and the canonical binding site is present in stable as well as unstable TTP-bound mRNAs.

In addition, there is a stronger impact of TTP-dependent mRNA degradation on the transcriptome of macrophages activated by lipopolysaccharide (LPS) during the early resolution phase of inflammation than during the onset of inflammation [13]. Inflammation is linked to the adaptive immune response since, following HLA-antigen complexes recognition, proliferating CD4^+^ T cells activate macrophages through cell-to-cell contact and IFN-γ secretion. TTP-mediated regulation of the HLA-DQ2 mRNAs stability is therefore proposed to occur during the resolution phase of inflammatory response. TTP interaction with these transcripts may counterbalance the high transcriptional expression of DQ2 genes and modulate the CD- and T1D-related antigen presentation. 

## 4. Conclusions

In previous work, it has been demonstrated that, in addition to HLA class II predisposing genotype, the high expression of risk alleles is relevant in the establishment of autoimmunity. In fact, the magnitude of the T cell activation and proliferation is related not only to the nature of the antigen but also to the amount of the antigen-HLA class II complexes expressed on the surface of APCs. The gene expression is mainly regulated at the transcriptional level, but RNA processing may also have an important role in the differential transcript synthesis. TTP/ZNFP36 RNA binding protein was identified as a member of RNP complex regulating the stability of HLA class II mRNA associated to CD and T1D. Most importantly, with the support of in silico approaches, it was revealed that the CD-associated mRNAs can strongly interact with TTP through their 3′UTR, conferring a more rapid mRNA turnover. These findings demonstrate that the synergy among molecular and in silico analysis unravel an allele-specific regulation. 

## 5. Materials and Methods

### 5.1. Cell Lines 

M14 human melanoma and human HOM cell lines (DQA1*01/DQB1*05 genotype) were obtained by ECACC (Sigma-Aldrich, Milan, Italy). B-LCL#5 are EBV-transformed B lymphoblastoid cell lines immortalized from PBMCs of celiac patients carrying DQA1*01-DQA1*05/DQB1*02-DQB1*05 genotype [9]. All cell lines were cultured in RPMI 1640 medium (Euroclone, Milan Italy)supplemented with 10% Fetal Calf Serum (Euroclone, Milan, Italy)). 

### 5.2. DNA Templates Synthesis and mRNA Quantification

The sequences of specific HLA alleles were obtained from the HLA database (http://www.ebi.ac.uk/ipd/imgt/hla/index.html) and the primers used for PCR and qRT-PCR were picked analyzing the sequences by Prime3web (http://primer3.ut.ee/) and then synthesized by Eurofins Genomics (Ebersberg, Germany). Some of the primers have been already used [9], and all sequences of are listed in Table 1. The template for 3DQA105 riboprobe synthesis was previously prepared [3], while the others obtained from retro-transcribed RNA. Specifically, we used cDNA from B-LCL#1 to obtain 3DB102 template and cDNA from HOM cell to synthetize 3DQA101 and DQB105 templates. Total RNA was prepared with the AurumTM Total RNA kit (BIORAD, Milan, Italy), and 1 µg of RNA was used for reverse transcriptase reactions, performed using an iScriptTM cDNA Synthesis kit (BIORAD). To quantify specific transcripts, we performed qRT-PCR using the Quanti Tect SYBR Green PCR Kit (BIORAD) through the DNA Engine Opticon Real-Time PCR Detection System (BIORAD). Each reaction was run in triplicate in the presence of 0.2 mM primers. The relative amount of specific transcripts was calculated by the comparative cycle threshold method [19], and GAPDH and β-actin transcripts were used for normalization. All results shown are the mean of at least three independent experiments. Statistical analysis was performed using the unpaired Student’s *t*-test with two-tailed distribution and assuming two samples equal variance parameters. In the figures, a single asterisk corresponds to *p* < 0.05 and double asterisks correspond to *p* < 0.01.

### 5.3. RNA Electrophoretic Mobility Shift Assay (REMSA) and Pull-Down 

The riboprobes synthesis and REMSA were performed according to the published protocol [3,4]. Briefly, the transcription reactions were performed using T7 in vitro transcription system (Ambion, Thermo Fisher, Milan, Italy) in presence of [^32^P] UTP (PerkinElmer, Milan, Italy) and riboprobes obtained were used in binding experiments with M14 and B-LCL#5 S100 extract. TTP recombinant proteins was produced as described [20].

For pull-down experiments, riboprobes were end-labeled with desthiobiotin cytidine and used in binding experiments with 60 µg of B-LCL#5 cytoplasmic extract with the Thermo Scientific Pierce Magnetic RNA-protein pull down kit (Thermo Fisher, Milan, Italy). The riboprobe used as negative controls was the 3′UTR of androgen receptor RNA poly(A)_25_ RNA, provided by the kit.

Desthiobiotinylated target RNAs bound to proteins were captured using streptavidin magnetic beads and, following washing and elution, the proteins interacting with RNA were separated by SDS-PAGE and analyzed by Western blot (Euroclone, Milan, Italy). We used three different antibodies, namely anti-DRBP76 (anti-double stranded RNA binding protein 76 or anti-NF90) antibody (BD Biosciences, Milan, Italy), N-terminus anti-EBP1 (Abcam, Cambridge, UK), and anti-TTP (Tristetraprolin, Santa Cruz Biotechnology, Dallas, TX, USA), to reveal the presence of proteins in the RNP complex binding 3′UTR. 

### 5.4. TTP Silencing and Phenotype Analysis

The plasmid for recombinant wild-type (AA) His-tagged TTP proteins (kindly provided by Dr. Tiedje) have been used to transfect HEK293 cells and protein purification using nickel-chelate agarose beads and following a protocol already described [20]. After imidazole elution, samples were dialyzed and stored at −80 °C in a solution made of 20 mM HEPES pH 8, 100 mM NaCl, 3 mM MgCl_2_, and 8% glycerol. 

For TTP depletion, we performed gene silencing using a pool of four different siRNA provided by Santa Cruz Biotechnology. In total, 5 × 10^5^ B-LCL#5 cells, transfected by nucleofector technology and Lonza kit, were harvested after 48 h either for protein extraction, flow cytometry analysis and RNA preparation. TTP depletion and overexpression were assessed by Western blot using an anti-TTP antibody. The HLA-DQ cell surface expression was performed by cytofluorimetric analysis using the FACSAria III and DIVA software with FITC mouse anti-human HLA-DQ antibody (BD Biosciences). The quantitation of specific transcripts was performed by qRT-PCR as described in [9], using primers listed in Table 1. 

## 6. Bioinformatics Analysis

Sequence alignments and identities were calculated using the global Needleman–Wunch algorithm [21], via EMBL-EBI API (https://www.ebi.ac.uk/Tools/psa/emboss_needle). Minimum Free Energy (MFE) RNA secondary structures were predicted using RNAfold [22] from single sequences. Structure accessibility probability scores were calculated using RNAplfold, [22] using a temperature of 37 °C, window size of 75 and mean structure score for fragments of 10 nt (RNAplfold -T 37 -W 75 -u 10) [13]. These parameters are similar to those used in the TPP Atlas describing transcriptome-wide TPP binding. In addition to MFE structure calculations, an ensemble of statistically sampled structures was generated using Sfold [15], for each riboprobe sequence indicating the most structured parts of the molecules common to the predicted ensemble clusters. To identify common structural motifs between the DQA1 and DQB1 sequences, we ran Foldalign [23], in a pairwise all-against-all search. Bracket notation secondary structure from RNAfold were rendered into images with selected motifs mapped onto them using FORNA [24].

AU rich motif mapping and enrichment analysis was performed with custom scripts, freely available at https://github.com/darogan/Tristetraprolin_Pisapia_DelPozzo including R code to recreate Figure 2. All possible kmers, for lengths 2, 3, 4, 5 and 6, were created for each of the riboprobe sequences, and the observed versus expected ratios calculated. Subsets of AU rich motifs are highlighted in the plots. We then mapped the same AU motifs onto each of the riboprobe sequences. Nested structures, i.e., overlapping, are indicated by the score on the y-axis of Figure 2.

## Figures and Tables

**Table 1:** List of primers.

**Primers Used for qRT-PCR**		
**Gene**	**Primers**	**Sequences 5′ → 3′**
β-Actin	ACT-F ACT-R	TCATGAAGTGTGACGTTGACA CCTAGAAGCATTTGCGGTGCAC
GAPDH	G-F G-R	AACGGATTTGGTCGTATTGGGC TCGCTCCTGGAAGATGGTGATG
HLA-DRA	DRA-F DRA-R	GGACAAAGCCAACCTGGAAA AGGACGTTGGGCTCTCTCAG
HLA-DRB1	DRB1-F DRB1-R	CTCAGCATCTTGCTCTTGTGCAG CAGCATTAAAGTCAGGTGGTTCC
HLA-DQA1	DQA1-F DQA1*R	GGTGTAAACTTGTACCAGT GGAGACTTGGAAAACACT
HLA-DQB1	DQB1-F DQB1*R	CAGATCAAAGTCCGGTGGTTT TCTGGGCAGATTCAGACTGAGC
HLA-A, -B, and -C	MHCI-F MHCI-R	AGTGGGCTACGTGGACGACA ATGTAATCCTTGCCGTCGTA
HLA-DQA1*05	alfa05-F alfa05-R	CGGTGGCCTGAGTTCAGCAA GGAGACTTGGAAAACACTGTGACC
HLA- DQA1*01	alfa01-F alfa01-R	CGGTGGCCTGAGTTCAGCAA GGAGACTTGGAAAACACTGTGACC
HLA- DQB1*02	beta02-F beta02-R	TCTTGTGAGCAGAAGCATCT CAGGATCTGGAAGGTCCAGT
HLA- DQB1*05	beta05-F beta05-R	ACAACTACGAGGTGGCGTACC CAGGATCTGGAAGGTCCAGT
**Primers used for PCR of riboprobes templates**		
**Probe**	**Primers**	**Sequences 5′ → 3′**
3DQA101	3DQA101T7 3DQA101R	TAATACGACTCACTATAGGCCATCCTGGAAGGGAAGTG TCAGGAGGTCAGGGAAAGAA
3DQA105	3DQA105T7 3DQA105R	TAATACGACTCACTATAGGATCCCATCCTGGAATGGAAGTG AAAGGCATTTGTGAGCTCTGAGCAG
3DQB102	3DQB102T7 3DQB102R	TAATACGACTCACTATAGGGGCACTGACTCCTGAGACT GCTGTGGGTCAGTGCAG
3DQB105	3DQB105T7 3DQB105R	TAATACGACTCACTATAGGGGCACTGACTCCTGAGACTGT GGCTGTGGGTCAGTGCAG

## Supplementary Materials

The following are available online at https://www.mdpi.com/2073-4409/8/12/1570/s1, Figure S1: Sfold Structure Comparison of 3′UTR of DQA1* and DQB1*. Each of the riboprobe sequences was analyzed with Sfold to predict statistical ensembles of structures. The ensembles permit many different conformations of the structures and clustering, indicating the most likely conformations adopted by each sequence. The minimum-free energy (MFE) structure is also predicted and its parent cluster identified. (**A**) 3DQA101; (**B**) 3DQA105; (**C**) 3DQB105; and (**D**) 3DQB102 show the ensemble structures to contain fewer conserved base pairings (grey boxes) than the MFE structure indicating the structure is more dynamic/flexible outside of these regions. This is particularly evident in (**D**) (3DQB102) where there is little conservation of base pairings in the large stem, as seen in the other structures, Figure S2: Sequence Alignment for human and mouse homologs for DQA1* and DQB1* alleles. The sequence alignments for the human DQA1* and DQB1* 3′UTR sequences in Figure 1A,B have been extended to include the mouse homologs H2-Aa and H2-Ab1. (**A**) The DQA1* alignment for the human alleles and mouse homolog show high sequence identity between the human alleles at 94.1% but a much lower identity between the human and mouse sequences (<60%) as summarized in (**B**). (**C**) The DQB1* alignment for the human alleles and mouse homolog show a low sequence identity of <45% between species but >90% for the human sequences as summarized in (**D**). The low level of conservation between human and mouse (<60% for DQA and <45% for DQB) will result in different locations of AU rich motifs and affect their presentation in single stranded RNA structure regions.
