# Supplementary material for: Tristetraprolin/ZFP36 Regulates the Turnover of Autoimmune-Associated HLA-DQ mRNAs
Source: Cells. 2019 Dec 4;8(12):1570. doi: 10.3390/cells8121570 (PMC6953012; doi:10.3390/cells8121570)
Supplement: Supplementary file 1 [file cells-08-01570-s001.pdf]

cells

Article

Tristetraprolin/ZFP36 Regulates the Turnover of Autoimmune-Associated HLA-DQ mRNAs

Laura Pisapia [1,†], Russell S. Hamilton [2,†], Federica Farina [1], Vito D'Agostino [3], Pasquale Barba [1], Maria Strazzullo [1], Alessandro Provenzani [3], Carmen Gianfrani [4] and Giovanna Del Pozzo [1,*]

[1] Institute of Genetics and Biophysics "Adriano Buzzati Traverso" CNR, Via Pietro Castellino, 111, 80131 Naples, Italy; laurapisapia@virgilio.it (L.P.); federica.farina@igb.cnr.it (F.F.); pasquale.barba@igb.cnr.it (P.B.); maria.strazzullo@igb.cnr.it (M.S.)

[2] Centre for Trophoblast Research, Department of Physiology, Development and Neuroscience, University of Cambridge, Downing Site, Cambridge CB2 3DY, UK; rsh46@cam.ac.uk

[3] Centre for Cellular, Computational and Integrative Biology-CIBIO, University of Trento, via Sommarive 9, 38123 Trento, Italy; vito.dagostino@unitn.it (V.D.); alessandro.provenzani@unitn.it (A.P.)

[4] Institute of Biochemistry and Cell Biology-CNR, Via Pietro Castellino, 111, 80131 Naples, Italy; c.gianfrani@ibp.cnr.it

* Correspondence: giovanna.delpozzo@igb.cnr.it

† Co-first authors.

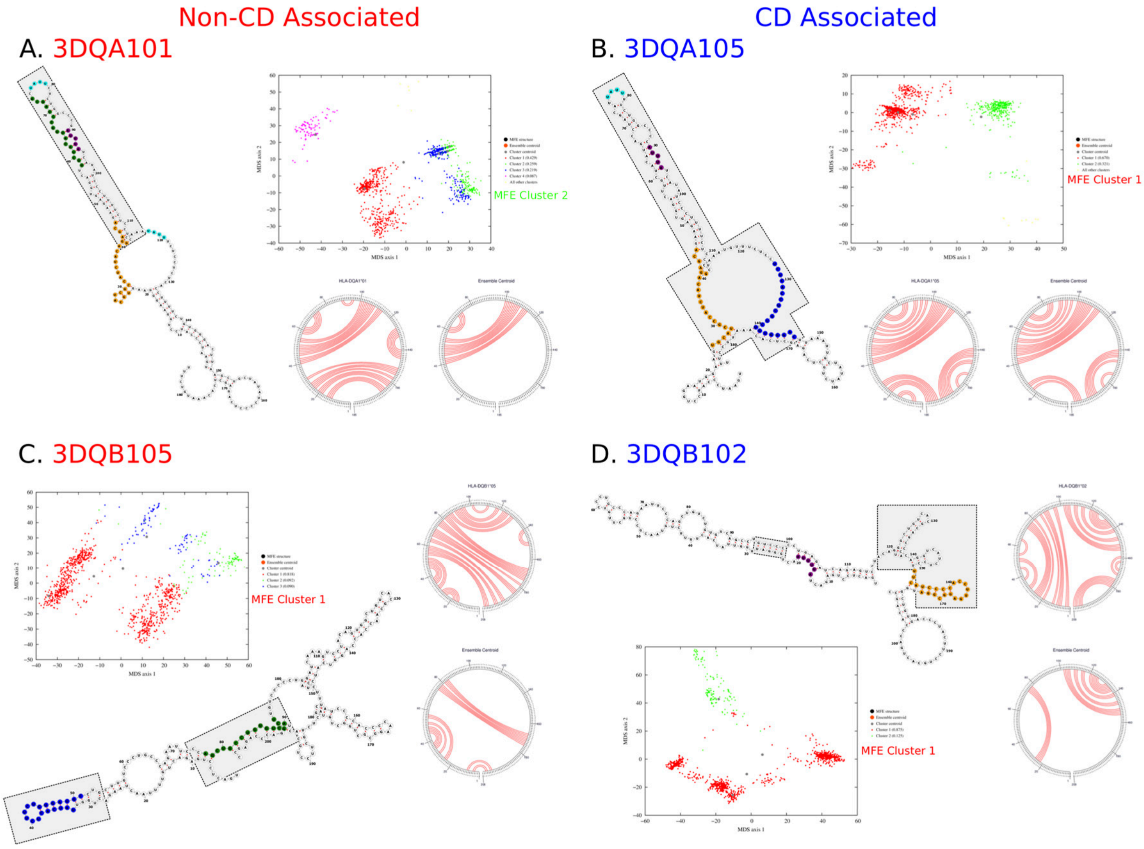

Figure S1. Sfold Structure Comparison of 3′UTR of DQA1* and DQB1*. Each of the riboprobe sequences was analysed with Sfold to predict statistical ensembles of structures. The ensembles permit many different conformations of the structures and clustering indicating the most likely conformations adopted by each sequence. The minimum-free energy (MFE) structure is also predicted and its parent cluster identified. **A.** 3DQA101, **B.** 3DQA105, **C.** 3DQB105 and **D.** 3DQB102 show the ensemble structures to contain fewer conserved base pairings (grey boxes) than the MFE structure indicating the structure is more dynamic/flexible outside of these regions. This is particularly evident in **D.** 3DQB102 where there is little conservation of base pairings in the large stem as seen in the other structures.

A.

```
3DQA101     UGAAUCCCAUCCUGGAAGGGAA-GUGCAUCGCCAUCUACAGGAGCAGAAGAGUGGACUUG
3DQA105     UGAAUCCCAUCCUGGAAUGGAA-GUGCAUCGCCAUCUACAGGAGCAGAAGAGUGGACUUG
H2-Aa-204   ---GUCACACCCUGGAAAGGAAGGCGUGUGUCCCUCUUCAGGGAAGAAGUGGUGUGCUGG

3DQA101     CU-ACAUGACCUAGCACUAUUCUCUGGCCCGAUUUAUCAUAUCCCUUUUCUCCUCCAAAU
3DQA105     CU-ACAUGACCUAGCAUUAUUUUCUGGCCCCAUUUAUCAUAUCCCUUUUCUCCUCCAAAU
H2-Aa-204   GUGACCUGGCACAGUG-UGUUUUCUGGACCAAUUCAUGGUGUUCUUUCUCUUCUUCAAGU

3DQA101     AUUUCUCCUCUCACCUUUUCUGUGGGACUUAAGCUGCUAUAUCCCCUCAGAGCUCACAAA
3DQA105     GUUUCUCCUCUCACCUCUUCUGUGGGACUUAAAUUGCUAUAUCUGCUCAGAGCUCACAAA
H2-Aa-204   GACCCCCAACUUGCUUUUCUCUUGACCCUGAGGCUGU----CCCUCUCACAGCUCACACA

3DQA101     UGUCUUU
3DQA105     UGCCUUU
H2-Aa-204   C-CCUUG
```

B.

	3DQA101	3DQA105	H2-Aa-204	GC
3DQA101		94.1	57.8	0.46
3DQA105	174/185		57.8	0.44
H2-Aa-204	108/187	108/187		0.52

C.

```
3DQB102     UGACUCCUGAGACUAUUUUA-ACUGGGA---UUGGUUAUCACUUUUCUG---UAACGCCU
3DQB105     UGACUCCUGAGACUGUUUUA-ACUAAGA---CUGGUUAUCACUCUUCUG---UGAUGCCU
H2-Ab1-201  UGACUCAGUUGACUGUCUCAGACUGUAAGACCUGAAUGUCUCUGCUCCGAAUUCCUGACU

3DQB102     GCUUGUC--CCUGCC---------CAGAAUUC--CCAGCUGUCUGUGU-----CAGCCUG
3DQB105     GCUUGUC--CCUGCC---------CAGAAUUC--CCAGCUGCCUGUGU-----CAGCUUG
H2-Ab1-201  GCCAGUCCACCUGCCACUCCGACUCAGAGUCUAGCAUGGUACUAUUGUAUCCACCACCUC

3DQB102     UCCCCCUGAGAUC-AGAGUCCUACAG----UGGCUGUCACGCAGCCACCAGGU-------
3DQB105     UCCCCCUGAGAUC-AAAGUCCUACAG----UGGCUGUCACGCAACCACCAGGU-------
H2-Ab1-201  AGCUCUUGUGAUCUGGAGUCCCCCAGUCUCUGUCUGUAGCUCUGCUCCUUGGUGAUUCCA

3DQB102     -------CAUCUCCUUUCAUCCCCACCUUGAGGCGGAUGGCUGUGACCC-----------
3DQB105     -------CAUCUCCUUUCAUCCCCACCCCAAGGCG-CUGGCUGUGACUC-----------
H2-Ab1-201  GAGACUCCAUCUGUGUACAGCCGCACCCCAGGCUUUCUGUCUUCGAUCUGUAGUAAACCA

3DQB102     -----UACUU-------CCUGCACU------------GACCCACAGCC
3DQB105     -----UGCUU-------CCUGCACU------------GACCCAGAGCC
H2-Ab1-201  AUGUAUGCUUAUCCCCACCUAGAUUACAAAUAAACGAGACUCAGACUC
```

D.

	3DQA102	3DQA105	H2-Ab1-201	GC
3DQA102		92.3	43.4	0.55
3DQA105	193/209		45.8	0.55
H2-Ab1-201	125/288	132/288		0.50

Figure S2. Sequence Alignment for human and mouse homologs for DQA1* and DQB1* alleles. The sequence alignments for the human DQA1* and DQB1* 3′UTR sequences in Figure 1A and B have been extended to include the mouse homologs H2-Aa and H2-Ab1. **A**. The DQA1* alignment for the human alleles and mouse homolog show high sequence identity between the human alleles at 94.1% but a much lower identity between the human and mouse sequences (<60%) as summarised in **B. C**. The DQB1* alignment for the human alleles and mouse homolog show a low sequence identity of <45% between species but >90% for the human sequences as summarised in **D.** The low level of conservation between human and mouse (<60% for DQA and <45%DQB) will result in different locations of AU rich motifs and affect their presentation in single stranded RNA structure regions.
